# Use of digital health technologies to examine subjective and objective sleep with next-day cognition and daily indicators of health in persons with and without HIV

**DOI:** 10.1007/s10865-021-00233-x

**Published:** 2021-08-09

**Authors:** Ni Sun-Suslow, Laura M. Campbell, Bin Tang, Arin C. Fisher, Ellen Lee, Emily W. Paolillo, Anne Heaton, Raeanne C. Moore

**Affiliations:** 1 Department of Psychiatry, University of California, 220 Dickinson Drive, St B (8231), San Diego, CA 92103-8231, USA; 2 SDSU/UC San Diego Joint Doctoral Program in Clinical Psychology, San Diego, CA, USA; 3 VA San Diego Healthcare System, San Diego, CA, USA

**Keywords:** Mobile health, Experience sampling, Ambulatory assessment, Smartphones, Actigraphy, Everyday functioning

## Abstract

Most previous studies investigating sleep’s association with health outcomes have relied on averaged sleep quality and laboratory-based health measures. This study examines the dynamic within-person relationships between subjective (Ecological Momentary Assessment) and objective sleep (actigraphy) on next-day cognition (subjective and objective), mood, and engagement in daily activities using linear mixed-effects regression modeling. Participants included 94 individuals (59 people with HIV, 35 HIV-) aged 50–74, assessed daily for 14 consecutive days/nights. Subjective and objective sleep were well correlated and were both associated with subjective ratings of cognition, but not objective cognition. Worse subjective sleep was associated with next-day lower happiness and higher depressed mood, and more pain, but was not related to next-day daily activities. Objective sleep was associated with next-day depressed mood and feelings of worry, and was positively associated with next-day television watching. Results provide evidence to support the utility of real-time assessment for sleep and functional outcomes that may lead to potential personalized interventions for individuals with and without HIV.

## Introduction

Poor sleep has been repeatedly associated with worse health outcomes, including greater cognitive deficits (Scullin & Bliwise, 2015), worse mood (Kahn et al., 2013), and decreased engagement in beneficial health behaviors (Grandner, 2017). Extant literature has predominantly examined the relationships between average sleep quality (assessed via self-report questionnaires or laboratory-based polysomnography) with laboratory-based cognitive performance or retrospective self-reported mood. With advances in digital health technologies such as smartphone-based ecological momentary assessment (EMA), mobile cognitive testing, and actigraphy, we have the ability to assess sleep unobtrusively in participants’ natural environments over multiple, continuous days, as well as examine the dynamic associations between sleep, cognition, mood, and next-day functional outcomes with greater accuracy and specificity. Understanding dynamic within-person relationships associated with sleep can build a foundation for potential personalized interventions, such as automated daily reminders, which may be particularly beneficial for groups with higher rates of sleep disturbance such as older adults (Ohayon, 2002) and those with comorbid chronic health conditions, such as HIV (Wu et al., 2015).

EMA is a data collection technique that allows for repeated assessment in participants’ lived environments (Shiffman, 2009). Smartphone-based EMA has been shown to be feasible and valid in multiple clinical and non-clinical populations including older adults and people with HIV (PWH) (Cain et al., 2009; Moore et al., 2017), and may be particularly useful in reducing retrospective memory biases that are common in traditional sleep and mood questionnaires (Van Den Berg et al., 2008). Additionally, it is difficult to examine daily fluctuations in cognition using laboratory-based neuropsychological testing, as this mode of administration often requires repeated travel to the laboratory or clinic, is expensive, and is unable to consider how daily activities and changes in the environment may impact cognition. Self-administered, mobile cognitive testing is able to address these shortcomings as it allows for brief, repeated assessments within a participant’s natural environment (Moore et al., 2017). Recent research has shown that coupling EMA with mobile cognitive testing, also known as ecological momentary cognitive testing (EMCT), can lend unique insights into the dynamic relationship between psychological factors and cognition (Moore et al., 2017, 2020a, 2020b). Lastly, similar to issues with cognitive testing within a laboratory or clinic setting, the use of wrist-worn actigraphy to assess objective sleep is advantageous over polysomnography due to its ability to capture sleep information over numerous days within a natural sleep environment (Martin & Hakim, 2011).

When examining the relationship between sleep and cognition, several studies suggest poor subjective and objective sleep are associated with worse subjective and objective cognitive functioning, and also higher risk of future cognitive impairment (Scullin & Bliwise, 2015; Yaffe et al., 2014). In one of the only studies to-date examining the dynamic association between sleep and cognition, Gamaldo et al. (2010) found less daily sleep time was associated with worse daily global cognition among older African Americans. However, this study involved eight in-person assessments (in the participant’s apartment or an empty room in a library or office) using traditional paper-and-pencil tests, limiting feasibility in a clinical setting. There have been two previous EMA studies involving PWH that have reported both objective and subjective sleep measures to be associated with objective laboratory-based cognition (Campbell et al., 2020a) and subjective cognitive complaints (Byun et al., 2016). However, these studies only examined between-person effects as the measures of objective and subjective cognition were only assessed within a laboratory at one timepoint. In fact, there are no studies among PWH (to our knowledge) that examine within-person dynamic relationships between sleep and cognition. Beyond cognition, however, decreased sleep quality has been repeatedly linked to worse mood outcomes within persons (Kouros & El-Sheikh, 2015), and vice versa (Kahn et al., 2013). Similarly, sleep has also been demonstrated to impact next-day pain ratings in both HIV- individuals and PWH (Kuerbis et al., 2019; Moldofsky, 2001), as well as next-day functional outcomes, including increased daytime sleepiness (Wibbeler et al., 2012), decreased overall productivity (Gingerich et al., 2018), and decreased engagement in cognitively demanding tasks (Engle-Friedman et al., 2003).

Due to limited research on the dynamic assessments of sleep and next-day functioning, this study examines within-person relationships between subjective (smartphone-based EMA) and objective (wrist-worn actigraphy) sleep quality on next-day cognition (measured both subjectively and objectively), mood, and engagement in daily activities. We hypothesized that worse subjective and objective sleep would both relate to poorer next day objective cognition, with objective sleep having a stronger association with sleep than subjective sleep. In addition, when compared to objective sleep, we hypothesized subjective sleep would be more strongly associated with subjective next-day indicators of health (mood, pain) as well as likelihood of engagement in next-day, cognitively-demanding (intellectual) activities. Of note, while we covary for HIV status, we did not specifically examine HIV interactions as sleep quality was relatively similar across groups and we hypothesize that differences in these relationships would be due more so to differences in sociodemographic and/or environmental factors rather than HIV itself.

## Methods

### Participants

Ninety-four individuals (PWH: n = 59, HIV-: n = 35) aged 50–74 participated in the Real-Time Mobile Assessment of Daily Functioning Among Older HIV-Infected Adults study within the HIV Neurobehavioral Research Program (HNRP) at the University of California, San Diego. Inclusion criteria for the study included: age 50 years or older, ability to provide written informed consent, and fluency in English. Exclusion criteria included: severe mental illness (e.g., schizophrenia), history of a non-HIV neurological disorder (e.g., stroke), brain injury with loss of consciousness > 30 min, and history of severe learning disability (i.e., WRAT-4 reading standard score < 70), or working regular night shifts. Participants with positive urine toxicology (exception of marijuana due to its long duration of detection) or alcohol breathalyzer on day of their laboratory visit were rescheduled. Additionally, 2 participants were excluded due to working regular night shifts. To alleviate participant burden, if participants had been enrolled in another study at the HNRP within the past six months, their neurobehavioral and neuromedical data were used for this study. The UCSD Institutional Review Board approved procedures prior to implementation, and all participants demonstrated decisional capacity (Jeste et al., 2007) and provided written informed consent.

### Ecological Momentary Cognitive Testing (EMCT)

At the initial laboratory-based visit, participants were each given one study smartphone (Samsung Galaxy smartphone with 4G Android Operating System) and a wrist-worn actigraphy device. Participants were also given a 20–30-min individualized tutorial on how to complete EMA surveys and mobile cognitive testing, followed by a standardized baseline neuromedical interview and a battery of neuropsychological assessments. Participants completed the 14-day EMA period, during which they completed up to four EMA surveys per day. Surveys assessed subjective sleep quality, subjective cognition, mood, pain, and engagement in daily activity. They were randomly delivered throughout the day approximately three hours apart, adjusted to each participant’s sleep–wake schedules. Following two random surveys out of four per day, participants were prompted to complete mobile cognitive tests (detailed below). During this time, participants were asked to wear a wrist-worn actigraphy device to capture objective sleep variables (detailed below). Smartphones and actigraphy devices were returned to the laboratory after the 14-day period. Participants were compensated for baseline and follow-up laboratory visits and $1 for each EMA survey completed (maximum of 56 surveys).

### Subjective and objective sleep

Subjective sleep (duration and quality) was assessed with the following questions from the first survey of the day: “How many hours of sleep did you get?” with five possible response options (0–3, 4–6, 6–8, 8–10, or 10 +), and “How restful was your sleep?” (visual analog scale from 1 = *Not at all restful* to 10 = *Very restful*).

To measure objective sleep, participants wore the ActiGraph GT9X Link device (Actigraph, Pensacola, FL) 24/7 on their non-dominant wrist for the duration of the 14-day EMA period only removed when the device could get wet (e.g., bathing). During this time, participants were also asked to record time in which they tried to fall asleep (i.e., in bed with the intention of trying to fall asleep) and when they woke up (i.e., out of bed with the intention to start the day) in a written daily log to determine number of minutes in bed. On the same daily log, participants recorded when and why they took the device off. The ActiGraph GT9X Link device has been previously shown to reliably detect sleep and wakefulness (Cole et al., 1992). If sleep logs were missing, sleep and wakefulness were manually determined from Actigraph data by a trained research assistant using methods detailed elsewhere (Full et al., 2019). The following objective sleep variables were examined: total sleep time (in hours) and sleep efficiency (i.e., total sleep time divided by total time in bed). Objective total sleep time was collected as a continuous measure and transformed to categories to mimic subjective hours of sleep responses (i.e., 0–3, 4–6, etc.).

### Subjective and objective cognition

Subjective cognition (i.e., self-reported forgetfulness and difficulty concentrating) was assessed at each EMA survey with the questions, “I feel forgetful…” and “I am having difficulty concentrating…,” both on a five-point scale from 1 = *Not at all* to 5 = *Very much*. Ratings of forgetfulness and difficulty concentrating were averaged each day to create daily scores for each person.

Daily objective cognition was assessed using the Mobile Color-Word Interference Test (mCWIT) and the Mobile Verbal Learning Test (mVLT), both of which participants completed a different version daily. The mCWIT is based on the Stroop color-word interference paradigm and is a test of executive functioning, specifically inhibition. Participants were asked to read the color of the font out loud. Their voices were recorded by the Smartphone and later scored. The total score on the mCWIT was the total amount of time (in seconds) it took to complete the task; please refer to Moore et al. (2020a) for psychometric properties of this task. One participant’s mCWIT data were excluded due to colorblindness, and two participants’ data were excluded because they completed all trials incorrectly (i.e., said the word rather than the color). The mVLT is a verbal learning test in which participants are presented 12 semantically-unrelated words over three trials (see Moore et al., 2020b for psychometric properties of the mVLT). At the end of each trial, participants were asked to say all words they were able to recall. The total score on this task was determined by the number of words correctly recalled over the three trials, which was scored by two independent raters. mCWIT and mVLT trials were excluded from analyses if raters suspected cheating (e.g., help from others).

### Next day mood, pain, and engagement in activities

Participants reported current level of happiness, depression, worthlessness, anxiety, and worry. Happiness (e.g., “I feel happy…”) was presented on a scale from 1 = *Not at all* to 5 = *Very much* at each EMA survey. Pain was assessed using the question “What is your pain level right now?” on a visual analog scale from 1 = *Minimal or no pain* to 10 = *Severe pain*. Mood and pain ratings were averaged for each study day. At each EMA survey, participants also reported the current activity in which they were engaging. For this study in particular, we examined passive leisure activities (e.g., “watching TV,” “resting”) and intellectual activities (e.g., “working,” “reading”). See Moore et al. (2017) for more details on how daily activities were assessed (Moore et al., 2017).

### Neurobehavioral and neuromedical assessment

All participants completed a comprehensive, laboratory-based neurocognitive assessment across seven neurocognitive domains, including verbal fluency, executive functioning, speed of information processing, learning, memory, working memory/attention, and motor. Cognitive deficit scores were calculated for each cognitive domain and averaged across the test battery to derive a global deficit score (GDS) ranging from 0 = *normal* to 5 = *severe impairment* (Blackstone et al., 2012). More details on the neuropsychological battery can be found in Moore et al. (2020a). Participants were also administered the Lawton-Brody Instrumental Activities of Daily Living (IADL) questionnaire, from which a participant was considered IADL-dependent if they reported a decline or need for assistance in two or more IADL domains (Lawton & Brody, 1969). Employment status was determined via self-report on the Patient’s Assessment of Own Functioning Inventory (Chelune et al., 1986). Psychiatric comorbidities were assessed using the computer-assisted fully structured Composite International Diagnostic Interview (Organization, 1998), which is consistent with DSM-IV diagnoses. At their baseline visit, participants also completed the Beck Depression Inventory-II (BDI-II) to assess current level of depressive symptoms (Beck et al., 1996).

Medical comorbidities were determined by self-report during a standardized neuromedical interview. A participant was considered to be using sleep medications if they reported being prescribed a medication approved by the US Food and Drug Administration for insomnia (i.e., butabarbital, doxepin, estazolam, eszopiclone, flurazepam, quazepam, ramelteon, secobarbital, suvorexant, tasimelteon, temazepam, triazolam, zaleplon, and zolpidem), trazodone, or reported taking over-the-counter insomnia drugs (i.e., diphenhydramine, doxylamine, and melatonin). The following HIV disease characteristics were determined via self-report: AIDS diagnosis, nadir CD4 (unless laboratory value was lower than reported nadir CD4), estimated duration of infection, and current and duration of treatment with antiretroviral therapy (ART) regimen. Viral load detectability (< 50 copies/mL) and current CD4 were measured in blood plasma. HIV and HCV serostatus were determined using an HIV/HCV antibody point-of-care rapid test (Miriad-Med-Mira^™^, Nova Scotia, Canada) and confirmatory Western blot analyses.

### Statistical analyses

Group differences by HIV serostatus in participant characteristics (e.g., demographics, HIV disease characteristics) were analyzed using Chi-Square test, Fisher’s exact test, and independent samples *t*-test as appropriate, and were presented as Cohen’s *d* effect sizes for continuous variables and odds ratio for binary variables. Objective total sleep time was continuous measures (range = 0.43–14 h) assessed by actigraph, which were categorized into 5 groups (the same as categorical subjective total sleep time, i.e., 0–3, etc.) in the analyses of determining the relationships of objective total sleep time with cognitive functioning, mood, and pain. The effects of subjective and objective sleep (i.e., total sleep time and sleep restfulness or efficiency) on objective (i.e., mCWIT and mVLT) and subjective (i.e., forgetfulness and difficulty concentrating) cognitive functioning were examined using separate linear mixed-effects models with subject-specific random intercepts, controlling for study day. Variables that we hypothesized may be associated with the outcome (i.e., HIV status, BDI-II total score, any lifetime substance use diagnosis and total number of sleep medications) were then included as covariates in the models of subjective cognition if *p* < 0.2; covariates were individually removed if > 0.2 starting with the covariate with the greatest *p*-value. Similarly, the relationships of subjective and objective sleep with mood (e.g., happiness, depression) and pain were assessed using linear mixed-effects model, adjusted for study day. HIV status, gender, and total number of sleep medications were then considered as potential covariates and retained in models if *p* < 0.2. In addition, the relationship between subjective and objective sleep and the frequency of next-day engagement in passive leisure or intellectual activities (possible range = 0–4 for each type activity) were examined using mixed-effects Poisson regression models with subject-specific random intercepts, controlling for study day. To improve normality of distribution and linearity of association, transformation was performed as appropriate, e.g., arcsine square root transformation was employed on objective sleep efficiency prior to the statistical analyses. Unadjusted *p*-values are interpreted in the results and discussion; however, unadjusted *p*-values that were corrected for multiple testing with the Benjamini-Hochberg (BH) procedure are also presented for analyses in Tables 2, 3 and 4. The significance level of alpha was set at 5%. All analyses were implemented using R version 3.6.0 (2019).

## Results

### Participant characteristics

Participant demographic and clinical characteristics are presented in Table 1. Our sample consisted, on average, of individuals in their late 50 s (PWH mean age = 59.3; HIV-mean age = 58.9), who had some college education, and predominately self-identified as Caucasian. PWH had a greater proportion of men than the HIV- group (83% versus 51%; *p* = 0.002, *OR* = 0.22); the groups did not differ on other demographic characteristics (age, education, ethnicity, premorbid IQ). Psychiatrically, when compared to HIV- individuals, PWH reported more current depressive symptoms on the BDI-II (*d* = 0.79, *p* < 0.001; with a median score within the minimal range) and were more likely to meet criteria for a lifetime Major Depressive Disorder (MDD; *OR* = 6.97, *p* < 0.001). The groups did not significantly differ on current or lifetime substance use diagnoses (*p*’s > 0.08). Most PWH exhibited evidence of ART-induced immune reconstitution, as indicated by active ART use (93%), undetectable HIV RNA viral loads (97%), and markedly higher current CD4 counts (median = 690 cells/mm^3^) compared to nadir CD4 counts (median = 196 cells/mm^3^). Mixed-effects models were performed to examine the effect of HIV status on sleep. HIV status was not significantly associated with subjective sleep restfulness (*p* = 0.17) or total objective sleep time (*p* = 0.44), but was marginally associated with objective sleep efficiency (*p* = 0.05), such that PWH had better sleep. It should be noted that categorical subjective hours of sleep were coded in a continuous fashion for these analyses.

### Comparing subjective and objective sleep measurements

**Fig. 1:**
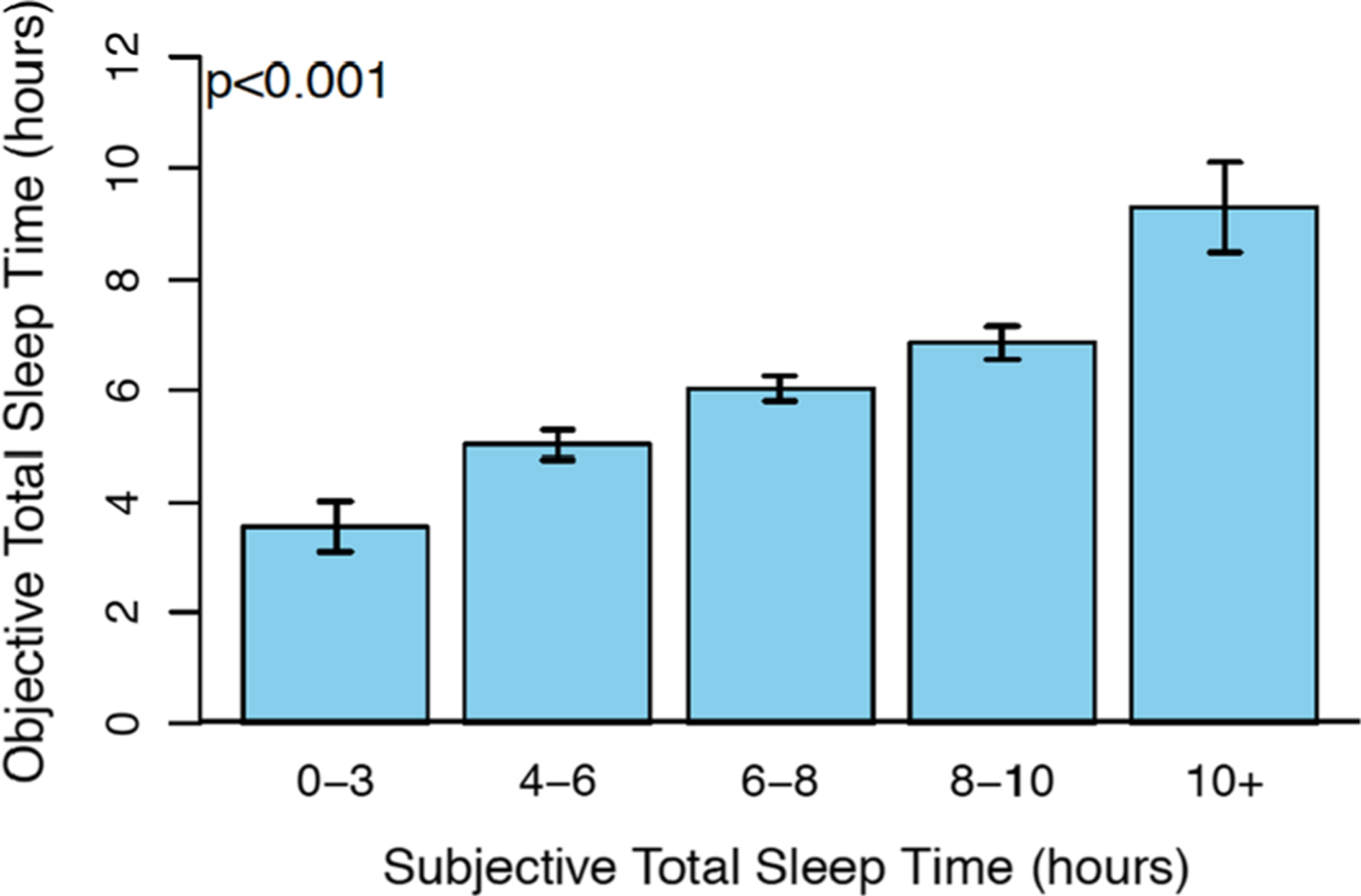
Objective total sleep time is associated with increased subjective total sleep time (*p* < .001)

Subjective sleep responses measured via EMA surveys were compared with objective actigraphy data. Subjective ratings of restfulness were significantly associated with objective measurements of sleep efficiency within persons (*B* = 0.056, 95% CI [0.042, 0.069], *p* < 0.001), such that for every 10% increase in sleep efficiency, there was a 0.56-point increase in subjective restfulness. Subjective restfulness was also associated with objective total sleep time within persons (*B* = 0.420, 95% CI [0.322, 0.517], *p* < 0.001), such that with every one-hour increase in objective sleep time there was a 0.42-point increase in subjective restfulness. Subjective total sleep time was also positively associated with objective total sleep time within persons (Fig. 1; χ^2^ = 314, df = 4, *p* < 0.001). In these analyses, subjective sleep was treated as a categorical variable with each response as a different group level (i.e., 0–3 h, etc.) and objective sleep was kept as a continuous variable.

### Subjective sleep and next-day cognitive functioning

The within-person relationships between subjective sleep and next-day cognitive functioning are presented in Table 2. Overall, subjective sleep ratings (total sleep time and restfulness) were associated with subjective next-day ratings of cognitive functioning (experiencing forgetfulness and having difficulty concentrating; *p*’s < 0.03), but not objective cognitive functioning (performance on mCWIT and mVLT; *p*’s > 0.21). Specifically, higher total reported sleep time and greater reported restfulness was associated with lower average daily rating of forgetfulness and lower average daily rating of having difficulty concentrating within persons. In comparison to 6–8 total hours of sleep (recommended for optimal health by American Academy of Sleep Medicine and Sleep Research Society(Consensus Conference et al., 2015), reporting 0–3 or 4–6 h related to higher subjective ratings of forgetfulness (*B* = 0.246, *p* < 0.001 and *B* = 0.066, *p* = 0.03 respectively), as well as greater reported difficulty concentrating (*B* = 0.288, *p* < 0.001 and *B* = 0.070, *p* = 0.04 respectively), and 4–6 h marginally significantly related to higher ratings after multiple testing correction with the BH procedure (*p* = 0.051 and *p* = 0.074 respectively). However, reporting more than 8 h of sleep (8–10 h or 10 + hours) per night does not appear to have additional benefits. In terms of objective cognitive functioning, subjective sleep was not associated with the time taken to complete the mCWIT (*p*’s > 0.36) nor the total number correct on the MVLT (*p*’s > 0.21). These relationships held upon controlling for BDI-II score, HIV status, any lifetime substance use diagnosis, and total number of sleep medications (range 0–1).

### Objective sleep and next-day cognitive functioning

The within-person relationships between objective sleep and next-day cognitive functioning is presented in Table 3. Objective sleep (both total sleep time and sleep efficiency) was not associated with objective cognitive performance on the mCWIT (*p*’s > 0.08) or the mVLT (*p*’s > 0.33). These relationships remained non-significant after controlling for BDI-II score, HIV status, any lifetime substance use diagnosis, and total number of sleep medications. When objective total sleep time was analyzed in a categorical fashion (i.e., 0–3, 4–6, etc.), sleeping 0–3 h, in comparison to 6–8 total hours of sleep, was associated with higher ratings of forgetfulness (*B* = 0.087, *p* = 0.03; *p* = 0.12 after accounting for multiple testing correction), and sleeping 0–3 or 4–6 h of sleep was associated with more difficulty concentrating (*B* = 0.160, *p* = 0.001 and *B* = 0.069, *p* = 0.03 respectively; *p* = 0.004 and *p* = 0.06 respectively after multiple testing correction). Objective sleep efficiency was not related to either subjective or objective next-day cognitive functioning (*p*’s > 0.11).

### Sleep and next-day mood and pain

The within-person relationships between sleep and next-day mood and pain ratings are reported in Tables 4 and 5. Self-reported total sleep time was associated with feeling depressed such that, compared to occasions when participants reported 6–8 h of sleep, reporting 0–3 h (*p* = 0.04), 4–6 h (*p* = 0.03), and 10 + hours of sleep (*p* = 0.05) were associated with higher ratings of depression and trended to be associated after multiple testing correction with the BH procedure (*p* = 0.067; Table 4). Self-reported total sleep time was not related to other ratings of emotional states or pain (*p’s* > 0.21). Higher subjective ratings of restfulness were associated with greater happiness (*B* = 0.020, *p* = 0.02) and with less depression (*B* = −0.018, *p* = 0.003), and pain (*B* = −0.043, *p* = 0.003). Compared to 6–8 h of objective sleep, 8–10 (*B* = −0.108, *p* = 0.04) and more than 10 h (*B* = −0.272, *p* = 0.04) of sleep was associated with increased likelihood of reporting depression the next day. Compared to 6–8 h of objective sleep, having 0–3 (*B* = −0.130, *p* = 0.004), 4–6 (*B* = −0.101, *p* = < 0.001), or 8–10 (*B* = −0.099, *p* = 0.04) hours of objective sleep was associated with increased likelihood of reporting being worried the next day. These *p*-values were adjusted for multiple testing with the BH method.

### Sleep and next-day engagement in daily life activities

Subjective sleep was not associated with the reported frequency of next-day activities (*p*’s > 0.12; Table 6). Objective sleep (total sleep time and sleep efficiency) was largely unassociated with the frequency of next-day engagement in passive leisure or intellectual activities; however, greater total sleep time was significantly associated with greater frequency of next-day television watching (*B* = 0.092, *p* = 0.001; p = 0.004 after multiple testing correction; Table 7).

## Discussion

Prior work has repeatedly linked poor sleep to worse health outcomes. However, the majority of these studies are limited to the laboratory/clinic assessment environment, are prone to retrospective memory biases, and largely do not consider intra-individual variability. Thus, our results suggest the use of dynamic assessment methodologies such as actigraphy and EMCT to characterize real-time cognition, mood, and engagement in activities as a function of sleep uniquely contributes to the existing literature.

We found subjective and objective sleep were well correlated with each other. At face value, this appears to be contrary to prior studies reporting discrepancies between subjective and objective sleep (Kaufmann et al., 2019; Rezaie et al., 2018). These previous studies, however, have relied on retrospective measures of subjective sleep requiring individuals to estimate their sleep quality over long periods of time (e.g., weeks to months), which is psychometrically different than our daily assessments of subjective sleep. For example, previous studies have shown that retrospectively assessed subjective sleep quality likely overestimates the number of hours slept and/or abnormalities captured via objective measures and may be influenced by a number of factors, including current psychopathology (Tsuchiyama et al., 2003), chronic illnesses (Happe et al., 2005), cognitive functioning (Bastien et al., 2003), age (Kay et al., 2015), and pain (O’Donoghue et al., 2009), among others. These previous findings suggest that such retrospective measures of subjective sleep are prone to recall error and response biases. In contrast, our current findings indicate that real-time (i.e., daily) assessment of subjective sleep via EMA reduces recall error and may be more sensitive to detect associations with other daily experiences.

While subjective and objective measurements were correlated in this study, their relationships with cognitive and mood outcomes varied. We found that both poorer subjective sleep (less reported total sleep time and lower reported restfulness) and poorer objective sleep (less total sleep time) were associated with worse subjective cognition (forgetfulness and difficulty concentrating). However, neither objective nor subjective sleep were related to performance on our objective cognitive tasks (mCWIT and mVLT), which is inconsistent with broad consensus that insufficient sleep leads to objective cognitive slowing, decreased alertness, more varied attention, and poorer memory (Killgore, 2010). It is possible that within the current study, cognitive changes affected by sleep were minimal and thus can only be captured subjectively, as our objective measures may not be sensitive enough to detect such subtle changes. This notion goes along with the broad literature reporting weak correlations between subjective and objective cognition (Mendonca et al., 2016; Tsutsumimoto et al., 2017). The lack of relationship between objective sleep and objective cognition could also reflect our crude objective sleep measures, as there has yet to be an “optimal” definition of objective sleep. For instance, it has been argued that variability in sleep (i.e., how atypical the previous night’s sleep) may play a critical role for next day cognition (Kaufmann et al., 2016). Future studies targeting broader objective cognitive domains as well as consideration of additional sleep variables such as atypicality of sleep may be helpful.

Of note, in a previous between-persons study, our group found subjective sleep (EMA) and objective sleep (actigraphy) differentially related to laboratory-based cognitive test scores. In particular, individuals with lower average objective sleep time demonstrated worse working memory, individuals with lower average objective sleep efficiency had worse learning performance, and individuals who reported worse average subjective sleep quality had worse executive function and working memory (Campbell et al., 2020a, 2020b). A recent validation of the mVLT task indicated that this measure captures learning (Moore et al., 2020b), yet we did not observe a dynamic within-person relationship between this task and objective sleep efficiency that we hypothesized. Our differential finding highlights the importance of understanding differences between within-person and between-person relationships. Although previous literature has established a strong between-person relationship between sleep and cognition (i.e., individuals with worse sleep *on average* show worse cognitive performance *on average*), our results suggest that acute changes in sleep efficiency do not significantly impact cognitive performance within persons. One positive clinical implication from our results is that minor fluctuations in objective total sleep time are not likely to influence day-to-day objective cognitive functioning among PWH. Instead, clinicians may want to focus on sleep interventions for PWH known to have consistently poor sleep on average and/or those with widely variable sleep.

Ratings of worse subjective sleep were associated with decreased happiness, increased depressed mood, and more pain, and worse objective sleep was associated with increased depressed mood and increased worry. Sleep disturbances and increased negative mood commonly occur together (Thomsen et al., 2003) and both have been linked to increased pain ratings (O’Brien et al., 2010). It may not be surprising that participants’ self-perceptions (sleep and mood) are related given that health and behavior perceptions, including sleep quality, have been linked to decreased quality of life and objective illness (Suls & Wallston, 2003). Overall, these results support the clinical use of daily subjective and objective sleep assessments in order to better assess and subsequently treat underlying, sleep-related causes of depressed and anxious mood. The use of technology (e.g., EMA) is also highly feasible and likely to promote better adherence to daily assessments in a clinical setting compared to paper and pencil diaries (e.g., like that which is used in cognitive behavioral therapy for insomnia [CBT-I]), as it prompts for a response rather than relying on patients to remember to fill out a form.

Although subjective sleep ratings were not related to any type of next-day activities, increased objective sleep time was associated with greater frequency of reported television watching. The directionality of this finding is supported by some studies that suggest disturbed sleep leads to less next-day engagement in cognitively demanding tasks (Engle-Friedman et al., 2003); however, other studies have argued a lack of clear directional link between sleep and next day activities. Mead et al. (2019) found physical activity did not predict the subsequent night’s sleep, however, longer than average total sleep time (for the individual) was associated with less physical activity the following day. Although additional studies are needed to examine whether our result is reliable across samples, the current found relationship between sleep and television watching may be clinically important, as it could also influence downstream effects on cognition. For example, previous studies from our group have shown that, among PWH, greater time spent watching television is associated with worse overall cognitive performance between persons, and that watching television is associated with worse real-time executive functioning within persons (Campbell et al., 2020b; Moore et al., 2017). In addition, our findings suggest that interventions focusing on increasing frequency of cognitively stimulating activities among individuals with cognitive impairment (e.g., Yuill & Hollis, 2011) may want to consider sleep as a possible barrier to engagement.

This study adds to the literature as the first to our knowledge to report within-person relationships between both objective and subjective sleep on outcomes of next-day cognition, mood and engagement in activities using EMCT. However, this study has some limitations. First, in order to directly compare subjective to objective sleep, we only included total objective sleep time and sleep efficiency, thus, our findings cannot be generalized outside of these constructs. Additional actigraphy sleep outcomes (e.g., sleep latency, sleep fragmentation, etc.) may inform other aspects of objective sleep on functional outcomes. Second, while sleep (both subjective and objective) did not differ by HIV status, previous literature suggests PWH face a variety of unique social, physical, and socioeconomic stressors that may influence sleep (Pellowski et al., 2013). A more in-depth investigation into these factors and how they may contribute to or alter our findings would be helpful in future studies. Lastly, not all findings remained significant after adjusting for multiple comparisons. Studies with larger sample sizes are needed in order to replicate these findings and examine other research questions (e.g., interactions) which require more power.

The current study provides evidence to support the use of EMCT to examine sleep and functional outcomes in real-time within individuals with and without HIV. Our findings reveal that subjective, rather than objective, sleep appears to have the strongest associations with next-day subjective cognition, mood, and pain ratings, which is consistent with a previous EMA/actigraphy study examining similar outcomes (Parsey & Schmitter-Edgecombe, 2019). At the current state, daily, self-reported sleep quality may be especially clinically beneficial in detecting changes within various health-related aspects, above and beyond assessed objective sleep measures. Further, dynamic within-person relationships between sleep (both subjective and objective) and cognition seems to differ from previous laboratory-based findings, suggesting possible utility of real-time associations to improve detection and treatment of sleep and/or cognitive problems. Overall, a more in-depth understanding of dynamic sleep and next-day indicators of health associations can lead to potential personalized and more effective interventions.

## Figures and Tables

**Table 1:** Participant characteristics at baseline by HIV serostatus (n = 94)

Variable	HIV-(n=35)	PWH (n=59)	Effect Size	95% CI	*P* value
Demographics					
Age (years), mean (SD)	58.9 (6.5)	59.3 (6.3)	.06	−.36, .48	.78
Education (years), mean (SD)	14.9 (2.5)	14.0 (2.5)	−.33	−.75, .08	.12
Sex (male), N (%)	18 (51.4)	49 (83.1)	4.54	1.62, 13.43	**.002**
Ethnicity, N (%)	–	–	–	–	.71
African-American	6 (17.1)	13 (22.0)	–	–	–
Hispanic	5 (14.3)	5 (8.5)	–	–	–
Caucasian	22 (62.9)	39 (66.1)	–	–	–
Other	2 (5.7)	2 (3.4)	–	–	–
Premorbid verbal IQ estimate^a^, mean (SD)	105 (16.1)	103 (14.9)	−.114	−.53, .30	.59
Employment status, N (%)	–	–	–	–	.14
Employed Full- or Part-Time	16 (43.2)	22 (30.1)	–	–	
Retired	11 (29.7)	20 (27.4)	–	–	
Permanently disabled	4 (10.8)	19 (26.0)	–	–	
Personal income, N (%)	–	–	–	–	**< .001**
< $35, 000 per year	19 (57.5)	64 (91.4)	–	–	
$35, 000–$74, 999 per year	9 (27.2)	4 (5.7)	–	–	
> $75, 000 per year	5 (15.2)	2 (2.9)	–	–	
Family income, N (%)	–	–	–	–	**.01**
< $35, 000 per year	57 (86.4)	15 (46.9)	–	–	
$35, 000–$74, 999 per year	3 (4.5)	8 (25.0)	–	–	
> $75, 000 per year	6 (9.1)	9 (28.1)	–	–	
Marital status, N (%)	–	–	–	–	**< .001**
Married/living in a marriage-like relationship	14 (37.8)	8 (11.0)	–	–	
Divorced/separated/widowed	16 (43.2)	22 (30.1)	–	–	
Never married	_7_ (18.9)	43 (58.9)	–	–	
Psychiatric and Substance Use					
BDI-II^b^, median [IQR]	2 [0, 4]	7 [2, 11.5]	.78	.37, 1.20	**< .001**
Lifetime major depression, N (%)	9 (25.7)	42 (71.2)	6.97	2.54, 20.70	**< .001**
Current any substance use disorder, N (%)	1 (2.9)	2 (3.4)	1.16	.06, 70.3	.99
Lifetime any substance use disorder, N (%)	17 (48.6)	40 (67.8)	2.21	.87, 5.74	.08
Comorbid Health Conditions/measures					
Hypertension, N (%)	14 (40.0)	37 (62.7)	2.50	.99, 6.52	.05
Hyperlipidemia, N (%)	15 (42.9)	39 (66.1)	2.57	1.01, 6.72	**.03**
Type 2 Diabetes, N (%)	9 (25.7)	11 (18.6)	.67	.22, 2.07	.44
HCV, N (%)	2 (5.7)	17 (28.8)	6.57	1.40, 62.7	**.007**
BMI, mean (SD)	31.1 (9.7)	27.6 (5.4)	−.48	−.90, −.06	**.027**
Neurobehavioral Function					
GDS^c^, mean (SD)	.41 (.41)	.43 (.40)	.06	−.37, .48	.80
Percent IADL Dependence, mean (SD)	21.1 (8.4)	18.7 (8.0)	−.30	−.71, .12	.17
PAOFI unemployment, N (%)	21 (61.8)	40 (70.2)	1.24	.54, 3.90	.49
HIV Disease Characteristics					
AIDS Diagnosis, N (%)	–	40 (67.8)	–	–	–
Current CD4 (cells/uL), median [IQR]	–	690 [563, 835]	–	–	–
Nadir CD4 (cells/uL), median [IQR]	–	196 [61.5, 427]	–	–	–
Undetectable plasma HIV RNA, N (%)	–	55 (96.5)	–	–	–
Estimated years of infection, mean (SD)	–	22.9 (7.7)	–	–	–
On ART, N (%)	–	56 (93.3)	–	–	–
Duration on all ART (months), mean (SD)	–	207 (87.9)	–	–	–

a Premorbid verbal IQ estimated using the Wide Range Achievement Test-Fourth Edition; BDI-II = Beck Depression Inventory-Second Edition; HCV = Hepatitis C; BMI = Body Mass Index; GDS = global deficit score; IADL = Instrumental Activities of Daily Living; PAOFI = Patient’s Assessment of Own Functioning Inventory; ART = antiretroviral therapy; CI = confidence interval; Student t-tests were used for continuous variables and Fisher’s exact tests were used for categorical variables; effect size is Cohen’s *d* for continuous variables and odds ratio for binary variables;

b log10,

c square-root transformed; significant group differences (*p* < .05) are in bold

**Table 2:** Mixed-effects models for the association of **subjective sleep** with next day cognitive functioning

Outcome	Model	Predictor	*B*	95% CI	*p* value	Adj *p* value	Overall P
Objective cognition							
Mobile color-word interference test (mCWIT), seconds to complete*	1	Subjective total sleep time	–	–	.73		.73
	2	Subjective sleep restfulness	−.001	−.004, .002	.36		–
Mobile verbal learning test (mVLT), total correct	3	Subjective total sleep time	–	–	.65		–
	4	Subjective sleep restfulness	.082	−.046, .211	.21		–
Subjective cognition							
Forgetfulness	5§	**Subjective total sleep time 0–3**	.246	.125, .367	**< .001**	**< .001**	
		**Subjective total sleep time 4–6**	.066	.008, .125	**.03**	.051	
		Subjective total sleep time 8–10	−.048	−.110, .015	.13	.15	
		Subjective total sleep time 10 +	.140	−.050, .331	.15	.15	**< .001**
		Total number of sleep meds	.285	−.050, .619	.10		
		BDI-II	.041	.026, .057	< .001		
		LT any substance use (ref. no)	.173	−077, .422	.18		
	6	**Subjective sleep restfulness**	−.011	−.022, −.001	**.03**	**.03**	
		Total number of sleep meds	.272	−.063, .607	.12		
		BDI-II	.041	.026, .057	< .001		–
		LT any substance use (ref. no)	.174	−.076, .424	.18		
Difficulty concentrating	7§	**Subjective total sleep time 0–3**	.288	.152, .424	**< .001**	**< .001**	
		**Subjective total sleep time 4–6**	.070	.004, .135	**.04**	.074	
		Subjective total sleep time 8–10	−.038	−.108, .031	.28	.37	
		Subjective total sleep time 10 +	.006	−.206, .219	.95	.95	
		Total number of sleep meds	.348	.039, .656	.03		**< 0.001**
		BDI-II	.048	.034, .062	< .001		
		LT any substance use (ref. no)	.223	−.007, .453	.06		
	8	**Subjective sleep restfulness**	−.013	−.025, −.002	**.02**	**.03**	
		Total number of sleep meds	.336	.029, .645	.04		
		BDI-II	.047	.033, .062	< .001		–
		LT any substance use (ref. no)	.227	−.003, .0457	.06		

Categorical subjective sleep predictors (i.e., Subjective total sleep time 0–3, etc.) and overall P presented only for significant Subjective total sleep outcomes (i.e., forgetful and difficulty concentrating); BDI-II = Beck Depression Inventory-Second Edition; HIV status, BDI-II total score, any lifetime substance use diagnosis and total number of sleep medications were included as covariates if *p* < .2 in the backwards model selection; LT = lifetime; Overall P = *p*-value for total sleep time;

§ reference group is Subjective total sleep time 6–8 h;

* log10 transformed; Adj *p*-values are corrected for multiple testing with the Benjamini-Hochberg (BH) method (1) in comparisons of Subjective total sleep time 6–8 vs. the other sleep time groups; (2) in association analyses between the related outcomes (i.e., forgetfulness and difficulty concentrating) and Subjective sleep restfulness; significant associations (*p* < .05) are in bold

**Table 3:** Mixed-effects models for the association of **objective sleep** with next day cognitive functioning

Outcome	Model	Predictor	*B*	95% CI	*p* value	Adj *p* value	Overall *P*
Objective cognition							
Mobile color-word interference test (mCWIT), seconds to complete*	1	Objective sleep efficiency	−0.010	−.079, .059	.78		–
	2	Objective total sleep time	−0.004	−.009, .001	.08		–
Mobile verbal learning test (mVLT), total correct	3	Objective sleep efficiency	1.396	−1.850, 4.636	.40		–
	4	Objective total sleep time	0.111	−.116, 337	.33		–
Subjective cognition							
Forgetfulness	5	Objective sleep efficiency	−.209	−.444, .026	.08		
		Total number of sleep meds	.262	−.088, .612	.15		–
		BDI-II	.050	.032, .068	< .001		
	6§	**Objective total sleep time 0–3**	.087	.007, .168	**.03**	.12	
		Objective total sleep time 4–6	−.003	−.059, .053	.92	.92	
		Objective total sleep time 8–10	−.047	−.134, .041	.30	.60	.09
		Objective total sleep time 10 +	−.032	−.227, .163	.75	.92	
		Total number of sleep meds	.257	−.093, .607	.15		
		BDI-II	.050	.032, .068	< .001		
Difficulty concentrating	7	Objective sleep efficiency	−.106	−.374, .161	.44		
		Total number of sleep meds	.305	−.010, .621	.06		–
		BDI-II	.053	.037, .069	< .001		
		LT any substance use (ref. no)	.215	−.031, .461	.09		
	8§	**Objective total sleep time 0–3**	.160	.068, .251	**.001**	**.004**	
		**Objective total sleep time 4–6**	.069	.006, .133	**.03**	.06	
		Objective total sleep time 8–10	.043	−.057, .143	.40	.53	
		Objective total sleep time 10 +	.032	−.190, .254	.78	.78	
		Total number of sleep meds	.308	−.010, .625	.06		**.02**
		BDI-II	.053	.037, .070	< .001		
		LT any substance use (ref. no)	.212	−.036, .459	.10		

Categorical objective sleep predictors (i.e., Objective total sleep time 0–3, etc.) and overall *P* presented only for significant Objective total sleep outcomes (i.e., forgetful and difficulty concentrating); BDI-II = Beck Depression Inventory-Second Edition; CI = confidence interval; HIV status, BDI-II total score, any lifetime substance use diagnosis and total number of sleep medications were included as covariates if *p* < .2 in the backwards model selection; LT = lifetime; Overall P = *p*-value for objective total sleep time;

§ reference group is Objective total sleep time 6–8 h;

* log10 transformed; Adj *p*-values are corrected for multiple testing with the Benjamini-Hochberg (BH) method in comparisons of Objective total sleep time 6–8 vs. the other sleep time groups; significant associations (*p* < .05) are in bold

**Table 4:** Mixed-effects model for the association of **subjective sleep** with next day mood and pain ratings

Outcome	Model	Predictor	*B*	95% CI	*p*- value	Adj *p* value	Overall P
Happy	1	Subjective total sleep time	–	–	.52		
		Total number of sleep meds	–	–	.08		–
		Sex (ref. male)	–	–	.03		
	2	**Subjective sleep restfulness**	.020	.005, .036	**.01**	**.02**	
		Total number of sleep meds	−.425	−.908, .059	.09		–
		Sex (ref. male)	.442	.050, .833	.03		
Depressed	3	**Subjective total sleep time 0–3**	.137	.009, .264	**.04**	.067	
		**Subjective total sleep time 4–6**	.068	.007, .130	**.03**	.067	
		Subjective total sleep time 8–10	−.009	−.075, .056	.78	.78	**.03**
		**Subjective total sleep time 10 +**	.203	.003, .404	**.05**	.067	
		HIV status (ref. HIV−)	.271	.004, .538	.05		
	4	**Subjective sleep restfulness**	−.018	−.029, −.007	**.001**	**.003**	
		Sex (ref. male)	.278	.012, .545	.04		–
Worthless	5	Subjective total sleep time	–	–	.76		
		Sex (ref. male)	–	–	.09		–
	6	Subjective sleep restfulness	−.002	−.008, .004	.44	.44	
		Sex (ref. male)	−.084	−.180, .012	.09		–
Anxious	7	Subjective total sleep time	–	–	.84		
		HIV status (ref. HIV−)	–	–	.03		–
	8	Subjective sleep restfulness	−.011	−.021, .000	.05	.075	
		HIV status (ref. HIV−)	.299	.027, .570	.03		–
Worried	9	Subjective total sleep time	–	–	.21		
		HIV status (ref. HIV−)	–	–	.02		–
	10	Subjective sleep restfulness	−.009	−.020, .001	.08	.096	
		HIV status (ref. HIV−)	.311	.049, .572	.02		–
Pain	11	Subjective total sleep time	–	–	.51		
		HIV status (ref. HIV−)	–	–	.04		
	12	**Subjective sleep restfulness**	−.043	−.068, −.019	**.001**	**.003**	
		HIV status (ref. HIV−)	.712	.028, 1.395	.04		–

Categorical subjective sleep predictors (i.e., Subjective total sleep time 0–3, etc.) and overall P presented only for significant Subjective total sleep outcomes (i.e., depressed mood; *p* < .05); overall P = *p*-value for Subjective total sleep time; reference group is Subjective total sleep time 6–8 h; Adj P, *p*-values are corrected for multiple testing with the Benjamini-Hochberg (BH) method 1) in comparisons of Subjective total sleep time 6–8 vs. the other sleep time groups; 2) in association analyses between the related outcomes (pain and mood, e.g., happy, worried) and Subjective sleep restfulness; significant associations (*p* < .05) are in bold

**Table 5:** Mixed-effects model for the association of **objective sleep** with next day mood and pain

Outcome	Model	Predictor	*B*	95% CI	*p* value	Adj *p* value	Overall *P*
Happy	1	Objective total sleep time			.63		
		Total number of sleep meds			.11		–
		Sex (ref. male)			.05		
	2	Objective sleep efficiency	−.366	−.733, .007	.05		
		Total number of sleep meds	.369	−.848, .109	.14		–
		Sex (ref. male)	.411	−.006, .827	.06		
Depressed	3	Objective total sleep time 0–3	.037	−.047, .120	.39	.49	
		Objective total sleep time 4–6	−.020	−.078, .037	.49	**.04**	
		**Objective total sleep time 8–10**	−.108	−.199, −.017	**.02**	.49	**.01**
		**Objective total sleep time 10 +**	−.272	−.475, −.070	**.01**	**.04**	
		HIV status (ref. HIV−)	.198	−.038, .434	.10		
	4	Objective sleep efficiency	−.058	−.302, .186	.64		
		HIV status (ref. HIV−)	.193	−.042, .428	.11		–
Worthless	5	Objective total sleep time			.79		
		Sex (ref. male)			.09		–
	6	Objective sleep efficiency	.056	−.084, .197	.43		
		Sex (ref. male)	−.096	−.206, .014	.09		–
Anxious	7	Objective total sleep time			.38		
		Sex (ref. male)			.05		–
	8	Objective sleep efficiency	.031	−.210, .273	.80		
		Sex (ref. male)	−.281	−.559, −.003	.05		–
Worried	9	**Objective total sleep time 0–3**	−.130	−.212, −.048	**.002**	**.004**	
		**Objective total sleep time 4–6**	−.101	−.158, −.045	**< .001**	**< .001**	
		**Objective total sleep time 8–10**	−.099	−.189, −.010	**.029**	**.039**	
		Objective total sleep time 10 +	−.161	−.358, .036	.11	.11	**.001**
		Sex (ref. male)	−.296	−.510, −.081	.008		
	10	Objective sleep efficiency	.062	−.176, .300	.61		
		Sex (ref. male)	−.296	−.511, −.080	.01		–
Pain	11	Objective total sleep time			.36		
		HIV status (ref. HIV−)			.09		–
					.16		
	12	Objective sleep efficiency	−.216	−.815, .384	.48		
		HIV status (ref. HIV−)	.676	−.089, 1.441	.09		–

Categorical objective sleep predictors (i.e., Objective total sleep time 0–3, etc.) and overall P presented only for significant Objective total sleep outcomes (i.e., depressed mood; *p* < .05); overall P = *p*-value for Objective total sleep time; reference group is Objective total sleep time 6–8 h; Adj P, *p*-values are corrected for multiple testing with the Benjamini-Hochberg (BH) method in comparisons of Objective total sleep time 6–8 vs. the other sleep time groups; significant associations (*p* < .05) are in bold

**Table 6:** Mixed-effects Poisson regression models for the association of **subjective sleep** with next day activities

Outcome	Model	Predictor	*B*	95% CI	*p* value	Adj *p* value
Watching television	1	Subjective total sleep time	–	–	.15	.30
	2	Subjective sleep restfulness	.035	−.001, .072	.06	.12
Intellectual Activities	3	Subjective total sleep time	–	–	.95	.95
	4	Subjective sleep restfulness	.015	−.024, .055	.44	.44

Outcomes are the number of incidences (0–4) reported for specific activity; Subjective total sleep time variable is categorical; Adj P, *p*-values are adjusted for multiple testing with the Benjamini-Hochberg (BH) method within each class of sleep quality (i.e., Subjective total sleep time and efficiency separately)

**Table 7:** Mixed-effects Poisson regression models for the association of **objective sleep** with next day activities

Outcome	Model	Predictor	*B*	95% CI	*p* value	Adj *p* value
Watching television	1	**Objective total sleep time**	.092	.035, .148	**.001**	.004
	2	Objective sleep efficiency	.560	−.272, 1.392	.19	.25
Intellectual Activities	3	Objective total sleep time	.008	−.060, .076	.82	.82
	4	Objective sleep efficiency	.632	−.299, 1.564	.18	.25

Outcomes are the number of incidences (0–4) reported for specific activity; Objective total sleep time variable is continuous; Adj *p*-values are adjusted for multiple testing with the Benjamini-Hochberg (BH) method in association analyses between the related outcomes (i.e., watching television and intellectual activities) and Objective total sleep time or efficiency; significant associations (*p* < .05) are in bold
